# First case of *Anaplasma platys *infection in a dog from Croatia

**DOI:** 10.1186/1756-3305-5-49

**Published:** 2012-03-09

**Authors:** Viktor Dyachenko, Nikola Pantchev, Hans-Joerg Balzer, Ariane Meyersen, Reinhard K Straubinger

**Affiliations:** 1 Institute for Infectious Diseases and Zoonoses, Department for Veterinary Sciences, Faculty for Veterinary Medicine, LMU Munich, Veterinärstraße 13, 80539 Munich, Germany; 2 IDEXX Vet Med Lab, Moerikestraße. 28/3, 71636 Ludwigsburg, Germany; 3 Small Animal Clinic, Hörder Bahnhofstraße 5, 44263 Dortmund-Hörde, Germany

**Keywords:** *Anaplasma platys*, *Babesia vogeli*, CRP, Infectious canine cyclic thrombocytopenia, Croatia

## Abstract

**Background:**

It is known that *Anaplasma (A.) platys*, the causative agent of infectious canine cyclic thrombocytopenia, is endemic in countries of the Mediterranean basin. However, few reports are available from the Balkans. This case report describes a dog, which was imported from Croatia to Germany in May 2010. One month later the dog was presented to a local veterinarian in Germany due to intermittent/recurrent diarrhoea. Diagnostic tests were performed to identify infections caused by *Anaplasma *spp., *Ehrlichia *spp., *Hepatozoon canis, Babesia *spp., *Leishmania *spp., *Borrelia burgdorferi *and/or *Dirofilaria immitis*.

**Findings:**

Haematological examination of a blood smear revealed basophilic inclusions in thrombocytes, which were confirmed as *A. platys *with a species-specific real-time PCR. Additionally, an infection with *Babesia (B.) vogeli *was also detected (PCR and serology). No specific antibodies against *Anaplasma *antigen were detectable. Although the dog showed no specific clinical signs, thrombocytopenia, anaemia and elevated C-reactive protein (CRP) were observed. Sequencing of a 1,348-bp partial ribosomal RNA gene revealed highest homology to *A. platys *sequences from Thailand, Japan and France.

**Conclusions:**

*A. platys *was detected for first time in a dog imported from Croatia. As the dog was also co-infected by *B. vogeli*, unique serological and haematological findings were recorded. Thrombocytopenia, anaemia and elevated values of C-reactive protein were the laboratory test abnormalities observed in this case. *A. platys *infections should be considered in dogs coming from Croatia and adjacent regions.

## Background

*Anaplasma platys *(formerly *Ehrlichia platys*) was first identified and described in 1978 in Florida (USA) as a *Rickettsia*-like, platelet-specific organism in dogs with infectious canine cyclic thrombocytopenia (ICCT) [1]. Based on morphology and serological cross-reactions with *Ehrichia canis*, the microorganism was first proposed as *E. platys *[2]. Sequencing and phylogenic analysis of the 16S rRNA gene and GroESL operon showed that the pathogen was related to *A. phagocytophilum *and *A. marginale*, which led to reclassification and designation as *A. platys *[3,4].

In dogs *A. platys *organisms infect peripheral blood platelets and form basophilic inclusions in the cells, so-called morulae, which contain one or more subunits [1,5]. Both, the appearance of the pathogen in the platelets and the following thrombocytopenia are cyclic [1]. The initial thrombocytopenias may develop primarily as a consequence of direct injury to platelets by replicating organisms. However, immune-mediated mechanisms of thrombocytopenia become more important in subsequent thrombocytopenic episodes [1]. The fraction of infected platelets decreases dramatically in successive parasitaemias, but the associated thrombocytopenic episodes remain severe [6]. In general, the infection is accompanied by unspecific and mild clinical manifestation including anorexia, depression, generalized lymph node enlargement, pale mucous membranes and elevated rectal temperatures [1,7-9]. Nevertheless, a severe course of *A. platys *infection with ecchymotic haemorrhagia was reported to be caused by a Greek strain [10]. The pathogen is assumed to be transmitted by *Rhipicephalus sanguines*, as in several studies *A. platys*-DNA was detected in this tick species and co-infections in dogs with *E. canis *and *B. vogeli*, two pathogens that share the same vector, reinforce this speculation [11-13]. The vector competence of *R. sanguineus*, however, could not be proven so far [14]. Currently, *A. platys *has been described in both American continents (USA [2], Venezuela [15], Brazil [16]), Asia (China [17], Thailand [12], Taiwan [18], Japan [19]), Australia [20] and Africa [21]. In Europe the occurrence of *A. platys *has been shown in Mediterranean countries: Italy [22], France [23], Spain [8], Portugal [24], Turkey [25] and Greece [10]. Here the first case of a presumed autochthonous *A. platys *infection is described in a dog from Croatia.

## Case report

A one-year-old male dog was imported from Croatia to Germany in May 2010 and, according to the owner declaration, has never been outside Croatia before. The dog was presented to a local veterinarian in Germany one month after the import due to intermittent/recurrent diarrhoea. Diagnostic tests for infections uncommon for the German area were requested (CBC with blood smear review, complete serum chemistry analysis as well as a "travel disease profile"). No abnormalities were found during the clinical assessment.

**Figure 1:**
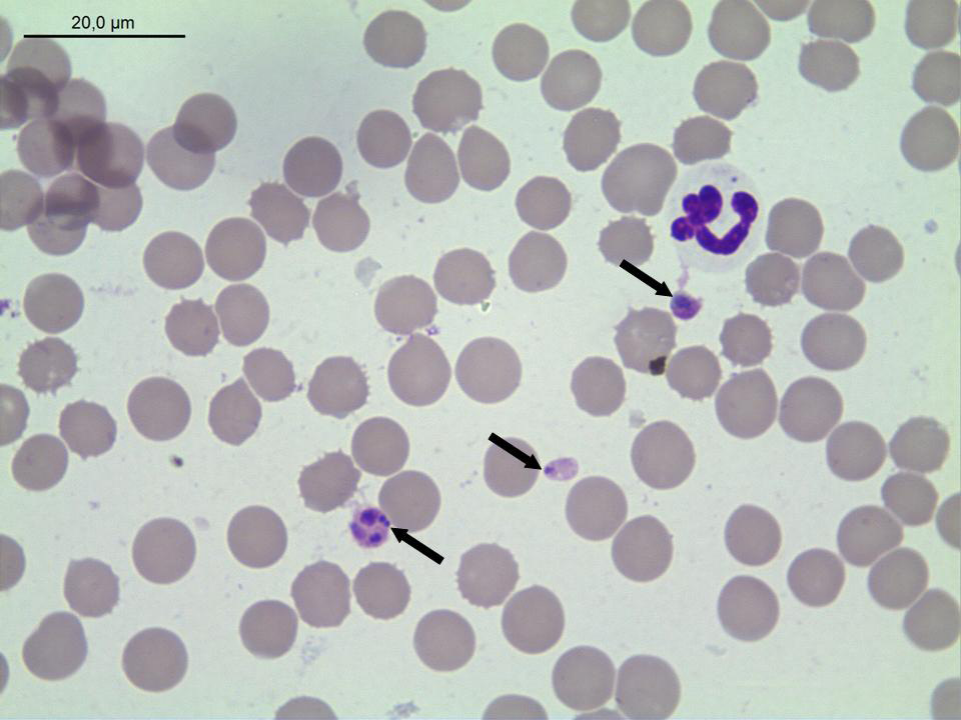
***A. platys *inclusion in blood platelets (arrows); Giemsa-stained blood smear of the dog presented in this report**.

Blood analysis indicated anaemia with erythrocytes at 4.20 T/L (reference range 6-9 T/L), haemoglobin of 9.4 g/dL (reference range 15-19 g/dL) and haematocrit of 32% (reference range 38-55%). The anaemia was classified as normocytic at the upper limit to macrocytic (MCV of 75 fL; reference range 60-75 fL) as well as hypochromic due to a mean corpuscular hemoglobin concentration (MCHC) lower than the reference range (30 g/dL; reference range 31-34 g/dL). Thrombocytopenia was registered as well (62 × 10^9^/L; reference range 150-500 × 10^9^/L). The differential and absolute white blood cell (WCB) counts were within the usual range (Table 1). Biochemistry parameters were within the reference range apart from a total protein at the lower limit of the reference range (53 g/L; 53-77 g/L), decreased albumin values of 2.70 g/dL (3.2-4.7 g/dL) as well as increased urea nitrogen of 34.7 mg/dL (10-25 mg/dL) and phosphorus at 2.0 mmol/L (0.7-1.6 mmol/L). A subsequent immunological examination revealed an elevated C-reactive protein (CRP, 38.2 mg/L; reference range 0-9.7 mg/L). In addition, the examination of a blood smear revealed basophilic inclusions in thrombocytes resembling *A. platys *(Figure 1). The following *A. phagocytophilum *and *A. platys-*specific PCRs confirmed the *A. platys *infection. The *A. platys*-positive PCR result (cycle threshold (Ct) value 19.1) was accompanied by a *B. vogeli*-positive PCR (Ct value 35.8). PCR testing for other Babesia (including *B. canis, B. rossi, B. gibsoni, B. conradae*), *Hepatozoon canis, Ehrlichia *spp. (including *E. canis, E. chaffeensis, E. ewingii) *and *Leishmania *spp. were negative. Serological assays as the SNAP 4Dx test (antibodies to *A. phagocytophilum, E. canis, Borrelia burgdorferi *and antigen of *Dirofilaria immitis*), microplate ELISA for antibodies against *Leishmania infantum *and IFA on *A. phagocytophilum, E. canis*, and *Leishmania*-antigen produced negative results. In contrast, a *B. canis-*specific ELISA was positive (a low level of antibodies at 20.2 test units was detected).

**Table 1:** Patient's haematological and serum biochemical parameters at the time of clinical examination

haematological parameters	found values	reference values	SI units
white blood cells	11.7	(6-12)	10^9^/L

red blood cells	4.20	(6-9)	10^12^/L

haemoglobin	9.4	(15-19)	g/dL

hematocrit	32	(38-55)	%

mean corpuscular volume	75	(60-77)	fL

haemoglobin E	22	(17-23)	pg

mean corpuscular haemoglobin concentration	30	(31-34)	g/dL

platelets	62	(150-500)	10^9^/L

basophil granulocytes	0	(0-1)	%
	
	0		

eosinophil granulocytes	1	(0-6)	%
	
	0.117	(0 - 0.6)	10^9^/L

neutrophil granulocytes (band)	0	(0-3)	%
	
	0	(0 - 0.3)	10^9^/L

neutrophil granulocytes (segmented)	70	(55-75)	%
	
	8.176	(3-10)	10^9^/L

lymphocytes	24	(12-30)	%
	
	2.803	(1-4)	10^9^/L

monocytes	4	(0-4)	%
	
	0.467	(0-0.5)	10^9^/L

atypical cells	1	(0)	

**serum biochemical parameters**			

protein	53	(53-77)	g/L

albumin	27	(32-47)	g/L

globulin	26	(15-35)	g/L

urea nitrogen	34.7	(10-25)	mg/dL

phosphorus	2.0	(0.7-1.6)	mmol/L

CRP	38.2	(0-9.7)	mg/L

creatinine	0.9	(< 1.4)	mg/dL

Based on abnormal clinicopathological findings in conjunction with the positive PCR results, specific therapy with doxycycline (10 mg/kg, orally, SID) was initiated for four weeks. After three weeks of Doxycycline therapy, a single injection of imidocarb dipropionate (6 mg/kg) was administered subcutaneously. In the following hours after the application the general health condition of the dog worsened, described as an anaphylactic reaction by the in-clinic veterinarian. Despite the immediate use of infusions and the administration of parenteral atropine, the dog died the next day.

## Methods

### Blood analysis

Blood was collected on the day of the presentation in the clinic. CBC with blood smear review was performed on the sample paying particular attention to blood parasites and hemotropic bacteria, as was complete serum chemistry (IDEXX Vet Med Lab). CRP concentrations were measured by means of a validated CRP immunoturbidimetric assay.

### DNA extraction and diagnostic PCRs

Total DNA was extracted from whole blood by using QIAamp DNA Blood Mini kit (QIAGEN, Germany) according to the manufacturer's instructions. Real-time PCR at IDEXX Vet Med Lab was performed using the LightCycler 480 (Roche) with proprietary forward and reverse primers and hydrolysis probes. Target genes for pathogen detection using real-time PCR were as follows: *A. platys *(groEL), *A. phagocytophilum *(msp2), *B. vogeli, B. canis, B. rossi, B. gibsoni *(hsp 70), *B. conradae *(ITS2), *H. canis *(18S rRNA), *Leishmania *spp. (GP63), *E. canis, E. chaffeensis, E. ewingii *(dsb).

### Serological examinations

Serological examinations for antibody detection were performed using an *A. phagocytophilum *IFA (MegaScreen FLUOANAPLASMA ph., cut-off = 1:50, MegaCor, Hoerbranz, Austria), a microplate *L. infantum *ELISA (*Leishmania*-ELISA Dog, Afosa GmbH Dahlewitz, Germany; reference range: negative < 7, borderline 7-12 and positive > 12), an *E. canis *IFA (MegaScreen FLUOEHRLICHIA c., cut-off = 1:40, MegaCor, Hoerbranz, Austria), and a microplate *B. canis *ELISA (*Babesia*-ELISA Dog, Afosa GmbH Dahlewitz, Germany; reference range: negative < 14, borderline 14-19 and positive > 19). In regard to *B. vogeli*, positive reactions were documented earlier utilizing a *B. canis *IFA test [26]. Furthermore, a rapid enzyme immunoassay assay test system (IDEXX SNAP^® ^4Dx^®^) was used as described elsewhere [27] following the manufacturer's directions. The IDEXX SNAP^® ^4Dx^® ^detects antibodies against *A. phagocytophilum *(p44), *Borrelia burgdorferi *sensu lato (C6), *E. canis *(p30, p30-1) as well as antigen of *Dirofilaria immitis*.

### Sequencing of 16S ribosomal RNA gene and sequence analysis

A 1,400-bp fragment of the 16S ribosomal RNA gene was amplified from total blood DNA by using forward (AGAGTTTGATCCTGGCTCAG) and reverse (CGGCTACCTTGTTACGACTT) Anaplasmataceae-specific primers, which were modified according to a study published elsewhere [28]. The reactions were prepared in total volumes of 50 μl with 15 pmol each primer, 0.2 mM each dNTP and 2 U Pwo DNA-polymerase in a 1× polymerase specific buffer complemented with MgSO_4_. After an initial denaturation step at 94°C the reactions cycled 30 times at 94°C for 30 sec, 58°C for 45 sec, 68°C for 90 sec and finally incubated at 68°C for 5 min in a Mastercycler pro (Eppendorf, Hamburg, Germany). The PCR products were purified with a peqGOLD Gel extraction Kit (PEQLAB, Erlangen, Germany) and submitted for sequencing to Eurofins MWG Operon (Ebersberg, Germany). The PCR product was sequenced three times in both directions. A 1,348-bp partial ribosomal RNA sequence was deposited to GenBank™ under accession number JQ396431.

## Results and discussion

With the exception of intermittent/recurrent diarrhoea the dog had no clinical signs, but anaemia and thrombocytopenia were evident in the haemogram (Table 1). Both are common, major abnormal clinicopathological findings observed in *A. platys *and *B. vogeli-*co-infected dogs [13] and are in concordance with the positive PCR results for *A. platys *and *B. vogeli *found in the dog. Infections with *A. platys *are difficult to detect, as in most cases the clinical manifestations are not clearly evident. *B. vogeli *infections usually cause subclinical to mild or moderate changes in adult dogs, however, concomitant conditions or additional pathogens present at the same time can exacerbate the course of infection [29].

Thrombocytopenia due to a monoinfection with *A. platys *has a cyclic character and is considered the result of the destruction of blood platelets by the proliferating pathogen during initial phase of infection, which probably triggers immunologic mechanisms in the subsequent course of the infection [6]. In *B. vogeli-*infected dogs, regenerative haemolytic anaemia is a well-known feature, but not all naturally infected dogs become anaemic. Furthermore, *B. vogeli *infections do not show a homogenous clinicopathological pattern [29,30]. Indeed, thrombocytopenia was reported to also be a haematological abnormality of *B. vogeli *infections, even though the decrease of platelets in blood is a consistent haematological abnormality in *B. canis *infections [30,31]. The thrombocytopenia can be enhanced by mixed *A. platys *and *B. vogeli *infections leading to significantly lower numbers of platelets compared to single pathogen infection [20]. Both, thrombocytopenia (2.5 fold below lower limit of normal range) and anaemia (low value of RBC count, haemoglobin and haematocrit), were found in the presented case (Table 1). Based on data generated with additional PCR and serology tests there was no evidence of co-infections with other pathogens such as *A. phagocytophilum, H. canis, Ehrlichia *spp. and *Leishmania *spp., which may occur also in Croatia and could take further influence on haematological parameters [32-34]. On the other hand, at the moment there are no available reports confirming the occurrence of *E. canis *in Croatia.

As representatives for acute phase proteins C-reactive protein (CRP, belonging to positive acute phase proteins) and albumin (negative acute phase protein) were evaluated, which in fact showed abnormal values. While CRP was elevated to up to 38.2 mg/L (reference range 0-9.7 mg/L), the serum albumin was decreased to 2.7 g/dL (reference range 3.2-4.7 g/dL) indicating systemic involvement likely due to a co-infection of *A. platys *and *B. vogeli*. Acute phase response as a part of the innate host defence system is linked to early innate responses for any pathological processes or diseases as reviewed elsewhere [35,36]. In dogs, CRP is known to show the highest response among acute phase proteins and has been used as an early unspecific marker [35]. An increase of CRP in dogs has been reported for many diseases caused for example by *B. canis *[31], *E. canis *[37], *L. infantum *[38] and *A. phagocytophilum *[39]. Thus far, no comprehensive investigations of acute phase response in tick-borne concurrent infections such as *A. platys *and *B. vogeli *exist.

The serological tests (IFA and SNAP 4Dx) based on *A. phagocytophilum *antigen failed to detect antibodies in the present case, but an ELISA based on *B. canis *antigen produced a positive result. However, the antibody level was low. It is known, that IFA tests with *B. canis *as a substrate cross-react with antibodies against *B. vogeli *and *B. rossi*, but homologous species antigens will cause stronger reactions [40]. Furthermore, that exact time point of seroconversion is not known for a natural *B. vogeli *infection and follow-up serological examinations after initial PCR-positive results, as shown previously [26], was not possible in the present case. The attempt to detect antibodies against *A. platys *was based on cross-reaction with *A. phagocytophilum *antigen. It has previously been shown, that the serum samples from naturally infected *A. platys *dogs from USA and China react positively with *A. phagocytophilum *antigen on the SNAP 4Dx [41,42]. Furthermore serum samples from *A. platys *infected animals in Portugal react well by means of IFA with *A. phagocytophilum *antigen [43]. Therefore, strain differences do not appear be a reason for the negative serological tests in the present study. According to the literature, seroconversion as a consequence of an *A. platys *mono-infection occurs between 13 to 19 days post infection as shown with strains collected in US and Greece [2,10]. As the dog was imported approx. 30 days before sampling to Germany and it is unlikely that the infection was acquired in Germany, an unusually long seronegative period can be inferred. It can be speculated that the tested blood samples were collected during the acute phase of infection probably before seroconversion occurred. This would be in concordance with negative IFA results as well as with high C-reactive protein and severe thrombocytopenia as described in *A. phagocytophilum*-infections previously [39]. The other possible reason for the seronegativity could be the dual infection. In case of a simultaneous infection with *A. platys *and *E. canis *it was documented, that *A. platys*-specific antibodies were detectable for the first time on average 27 days (range 14-35 days) post infection [42]. Consequently, the concurrent infections of *A. platys *and *B. vogeli *might have induced a delay in the humoral immunological response in this patient.

Intermittent diarrhoea was a reason for the presentation of the dog. The possible cause for the diarrhoea remains unknown, as no faecal sample was examined. But, it seems unlikely that intermittent diarrhoea alone could have led to hypoalbuminemia in the dog due to substantial gastrointestinal loss of fluids.

The dog died shortly after imidocarb dipropionate administration. However, the cause of death stays speculative, because no post-mortem examination was performed. An anaphylactoid reaction was observed in this case. Such reactions were described after imidocarb dipropionate administration in rare cases [44], while side effects of the drug (hypotension, hypersalivation, nasal discharge, lacrimation, diarrhoea, vomiting) can be reduced by atropine pretreatment. Furthermore, occasional renal tubular necrosis and hepatotoxicity after treatment have been described also [45,46]. In this case the elevated urea nitrogen of 34.7 mg/dL (reference range 10-25 mg/dL) and creatinine within the reference range (0.9; reference range < 1.4 mg/dl) are indicative for azotaemia but not for a strong renal involvement, which could exacerbate the side effects of imidocarb dipropionate therapy. In a group of seven dogs with *B. canis *infection, which had been preselected due to renal involvement and treated with imidocarb dipropionate, four dogs died spontaneously and the kidneys of all animals of the group showed degenerative changes of mainly the proximal convoluted tubules as well as necrosis of the whole tubule in some cases [47]. The authors reported that the histological alterations seen in the dogs were similar in dogs treated with imidocarb dipropionate and in the untreated animal. Hence, the pathological changes observed in the kidneys cannot be explained exclusively by the potential nephrotoxicity of imidocarb dipropionate [47]. In the case presented here it remains open, whether the dog died because of the side effects or as a result of the combined impact of the observed *B. vogeli *and *A. platys *infections. Nevertheless, it is recommended to treat simultaneously even mildly azotaemic dogs with proper intravenous fluid therapy when imidocarb dipropionate is applied and lower the dose of the drug (e.g. 3 mg/kg) in patients suspected to have renal involvement, in order to decrease the risk of renal insufficiency [47].

Sequencing of the 16S rRNA gene produced a 1,348-bp sequence, which was identical to other *A. platys *16S rRNA sequences generated in Thailand (EF139459), Japan (AY077619) and France (AF303467). The analysis of 16S rRNA sequences confirms the previous results, in which little genetic diversity was observed in 16S rRNA sequences of *A. platys *strains from different geographic locations [8,22,48].

The mixed *A. platys *and *B. vogeli *infection is well known from previous reports and represents a large fraction of co-infections among tick borne pathogens in dogs [20,26,49]. The examination of free-roaming dogs associated with remote Aboriginal communities in Australia showed 11% of dogs co-infected with both *A. platys *and *B. vogeli *[20]. The occurrence of *B. vogeli *in Croatia and other southern European countries is well documented [32]. The natural occurrence of *A. platys *in moderate climate zones and simultaneous coinfections with *Babesia *spp. allow the assumption that *Rhipicephalus *sp. e.g. *Rhipicephalus sanguineus *ticks may serve as the natural vector. On the other hand, this tick species has a wide genetic diversity [50] making it difficult to find the definitive vector for *A. platys*. The detection of *A. platys *in non-engorged questing adult *Rhipicephalus turanicus *tick in Israel [51] raises the question, whether other *Rhipicephalus *species actually serve as vectors for this agent.

To our knowledge this is the first report of *A. platys *infection in a dog imported from Croatia. Based on the owner's declaration, the dog has never travelled abroad before importation to Germany and the dog was presumably infected in Croatia. The infection in Germany is unlikely due to the following reasons: the vectors *Rhipicephalus *spp. ticks are commonly not present in Germany, even though ticks imported from abroad with travelled dogs can survive in homes with moderate temperatures [52]. The dog owners in Germany, however, did not report any tick infestation and so far there are no reports of autochthonous occurrences of *B. vogeli *and *A. platys *from the region where the dog spent its final days.

## Conclusions

*A. platys *infection was detected for the first time in a dog imported from Croatia. A co-infection with *B. vogeli *probably induced the untypical serological results. The major clinical manifestations were thrombocytopenia, anaemia and elevated values of C-reactive protein. *A. platys *infection should be considered in dogs living in or returning after travel from this area and showing abnormal clinicopathologic findings described in this report.

## Abbreviations

ICCT: Infectious canine cyclic thrombocytopenia; CRP: C-reactive protein; IFA: Immunofluorescence antibody assay; WCB: White blood cell; ELISA: Enzyme linked immunosorbent assay; Ct: Cycle threshold; CBC: Complete Blood Count; SID: Once daily.

## Competing interests

The authors have no competing interests.

## Authors' contributions

VD wrote the manuscript and performed sequencing and analysis of 16S rRNA. NP performed serological and clinicopathologic examinations, case consultation and correction of manuscript. H-JB performed diagnostic PCRs and correction of manuscript. AM performed anamnesis, clinical examination and treatment. RKS supervised and revised the manuscript. All authors read and approved the final version of the manuscript.
